# Mr. Taylor on Poisons in Relation to Medical Jurisprudence and Medicine

**Published:** 1848-07-01

**Authors:** 


Art. V.—*On Poisons, in relation to Medical Jurisprudence and Medicine.* By Alfred S. Taylor, F.R.S. Churchill, 1848. pp. 855.

Toxicology, as a distinct and important branch of medical jurisprudence, comes within the scope of the original design of this publication. Many circumstances contrive to direct attention to the subject. The great increase of late years in the crime of poisoning, and the frequency with which he is called upon to give his evidence on the point in a court of justice, not to mention the promptitude with which he is required to act at the bedside of the patient, render the study of this branch of science essential to the general practitioner. By those having the care of the insane also, it should be carefully investigated, as it affords much curious and useful information bearing upon their particular department. Poisoning among lunatics is rare, but still it is occasionally one of the forms of suicidal or homicidal mania. The perverted ideas and hallucinations respecting it, the dread of poison, and the belief that it is being constantly administered, are perhaps among the most common delusions of the alienated. To compare these imaginary sufferings with the real symptoms of poisoning, and from thence to deduce a common ailment of the internal organs in both cases—some morbid change or derangement of the *primæ viæ*—is both philosophical and useful. Toxicology shows us, likewise, the intimate connexion between mind and matter; that many medicines act exclusively on the nervous system, through the stomach, and when in deleterious excess, produce delirium, temporary or permanent mania, convulsions, coma, and paralysis. Finally, in proving that symptoms of poisoning result from the consumption of ordinary articles of food in certain states and conditions, toxicology teaches us the necessity of attending strictly to the diet of the insane, in order to restore the *mens sana in corpore sano.*

The progress of collateral sciences, and the rapid accumulation of fresh facts and observations, render a new work on poisons much to be desired. We are never satisfied unless we have the very latest information for our guidance. Mr. Taylor's work supplies this desideratum. It is a complete, and the best class-book on the subject of toxicology. It is written in a clear and lucid style, with ample reference to the sources of facts and opinions; moreover, it is an exceedingly amusing volume, and will be perused with pleasure by the general reader.

Mr. Taylor first treats of the medical evidence in cases of poisoning, and defines accurately the meaning of the word, "deadly poison," as distinguished from that which may become "a noxious thing" under peculiar circumstances. "The term *deadly* can be used with respect to those poisons only which may prove speedily fatal in small doses, such as strychnia, morphia, prussic acid, and arsenic; but that it could not with any sort of propriety be applied to such substances as sulphate of copper, of zinc, or spirits of hartshorn." After all, the word *deadly* must be regarded as an unnecessary adjunct—a surplusage. After classifying the different kinds of poison, he gives us some curious particulars under the head of *animal irritants.* Speaking of cheese and sausages, he says, (page 552,) "These articles of food have frequently given rise to symptoms of poisoning in Germany, but there is, I believe, no instance of their having proved fatal in England. The symptoms produced by cheese have been those of irritant poisoning. The nature of the poison is unknown. In examining several specimens of decayed cheese, I have found in them only an acrid oil, and sesquicarbonate of ammonia. In some cases the poisonous property is undoubtedly due to a putrefied state of the curd. Again, it has been supposed that the poison is occasionally derived from certain vegetables on which the cows feed. The symptoms caused by the *sausage poison* are very slow in appearing; sometimes two, three, or four days elapse before they manifest themselves—they partake of the narcotic irritant character. This poison is of a very formidable kind. In the "Medical Gazette," for Nov. 1842, there is an account of the cases of three persons who had died from the effects of liver sausages, which had been made from an apparently healthy pig, slaughtered only a week before. The inspection threw no light on the cause of death. The poisonous effect is supposed to depend on a partial decomposition of the fatty parts of the sausages. It is said, that when extremely putrefied, they possess no poisonous properties. Does not the same observation hold good with respect to dissection wounds?

Sometimes fatal effects follow the use of ordinary articles of animal food in a diseased condition. With regard to putrefaction, Dr. Taylor says (p. 555,) "it would appear that the flesh of the most healthy animal is rendered unwholesome by this process; but that the most severe effects are produced by that flesh which has become only partially decomposed; when, in short, putrefactive fermentation has been recently set up. The flesh of animals over driven, as well as newly killed meat in general, is liable to produce violent gastric irritation, and even cholera." In confirmation of this opinion, the following circumstance is stated to have occurred recently in the Grand Duchy of Baden:—"A roebuck, having been taken in a net, was killed while making violent efforts to escape, and while in a state of the utmost terror and exhaustion. Nearly all the persons who partook of the flesh of this animal experienced a violent gastro-intestinal inflammation, with other symptoms similar to those detailed above; although, in this instance, the flesh was neither in a putrefying state, nor were any of the cases fatal."

The influence of the mind upon the wholesomeness of the mother's milk is generally admitted. The following extraordinary instance is reported:—"A woman while suckling her child, became violently excited by the loss of some article which had been stolen from her. She gave her child the breast while in a state of violent passion. The child at first rejected it, but subsequently took a quantity of milk. Soon afterwards violent vomiting supervened. In the course of some hours the child took the other breast, when it was attacked with violent convulsions, and died in spite of medical aid."

On occasions when symptoms of irritation have been observed to follow the use of *cow's milk*, it would appear as if the secretion had really acquired a specific poisonous action. This must be frequently owing to the food of the animal, for Dr. Taylor observes, (p. 563,) "It is generally admitted that milk may become poisoned when the cow feeds upon *hyssop, spurge,* and other irritant vegetables; and this form of poisoning is well known to occur in other cases in which the cause is not so apparent. In the Dictionary of Natural History, a case is related where a patient was advised by his medical attendant to drink the milk of a cow fed on hemlock. The animal became emaciated, lost its milk, and fortunately for the patient, died from the effects of the poison, or it is not improbable he might have fallen a victim to this plan of treatment. The secretion easily undergoes changes, according to the food of the animal. It is rendered bitter when the cow feeds on wormwood, or sow thistle, the leaves of the artichoke, and its taste is affected by the cabbage, carrot, and all strong smelling plants. It is singular that the animal poison of rabies should be sometimes transmissible by the milk." We remember that some years back a medical man wrote a pamphlet to prove that most human ailments were caused by drinking the milk of cows fed on butter-cups. The absurdity of this hypothesis is certainly lessened by the above facts.

We now come to hydrophobia, a disease of the nervous system which is even yet but little understood, but with regard to which there are strange misconceptions abroad. The following particulars furnished by Mr. Taylor will be read with interest, although they should be given *in extenso* to do the laborious author justice.

It is singular that the dread of water, which is the main pathognomonic character of rabies in the human subject, is not met with in the animals which impart it. Rabid dogs commonly have an irresistible thirst, and drink water very readily. The danger of allowing dogs to lick the person is not only a disgusting but a dangerous practice; as it is impossible to say whether the saliva of an animal be or be not in a morbid condition. The Hon. Mrs. Duff died of hydrophobia from allowing a French poodle to lick her face whereon there was an abraded pimple. Dr. Colles met with the case of a young girl where the contact of the saliva of a rabid dog with the fine skin of the lips, led to a fatal attack of the disease. The effect of the poison upon the mental faculties is highly interesting. According to the researches of Mr. Taylor, the first signs of the disorder are headache, languor, and general lassitude. The senses of the patient are morbidly acute. He dislikes the smell of any familiar substances, and the reflection of light from polished surfaces. His pupils are dilated, and his countenance expressive of timidity and great anxiety. Sometimes he has been observed to shrink under the bed clothes in the most dreadful state of fear. The first symptom in the Duke of Richmond was, that early in the morning his valet found him alarmed at the appearance of some trees which were near to a window of the room where he slept, and which he insisted were people looking in. The respiration is humid and gasping; there is a suspicion of those about him, and he feels a sense of oppression at the epigastrium. With the exception of these signs of nervous excitement there is no other mental disturbance. The patient makes no attempt to injure those about him. There is no madness, the mind generally remains clear until the last, and there is very seldom delirium.

We must bring our notice of Mr. Taylor's excellent little volume to a conclusion, by examining his opinions on the subject of ether vapour, which, with other agents of a similar character, have been so extensively used lately for the production of insensibility during surgical operations. The remarks are so sensible and concise, that we find a difficulty in curtailing them. He says, p. 736, "It has been long known that the vapour of ether acted on the system as a powerful narcotic. The poisonous effects of the vapour have been known for a long time, although the attention of the profession has been only of late particularly drawn to the subject. . . . . . Ether, it is well known, gives off a heavy vapour, which possesses a strong odour at all temperatures. It is exceedingly diffusible and volatile, properties which are more favourable for the operation of this liquid in the state of vapour, than for the action of alcohol. When the vapour is respired, it enters the blood in the pulmonary vessels, and the effects are almost immediate. The individual falls into a lethargic condition, the respiration becomes slow, deep, and loud, the skin pale and cold, the lips assume a darker hue, the pulse is quickened, the eye is glassy, and the pupil dilated; the whole body is flabby and relaxed. A small quantity of ether introduced into the blood through the lungs, produces these striking symptoms in from two to four minutes; and if hot air be substituted as soon as unconsciousness begins, they disappear just as rapidly. In a more advanced stage, the pulse slackens, and the temperature of the body rapidly falls. Half an ounce of ether, or even less, inhaled in the form of vapour, would produce a much more powerful effect on the system, than one or two ounces taken into the stomach as a liquid. The sudden cessation of the sympathies, and the restoration of sensibility, are owing to the rapid elimination of the vapour through the lungs. If the respiration of the vapour be prolonged for from ten minutes to half-an-hour, there is coma, the pulse sinks, and there is some difficulty in rousing the individual. The after effects are also more serious: there is exhaustion, a feeling of stupefaction, with other unpleasant narcotic symptoms; but occasionally the patient has fallen into a quiet sleep. . . . . . As a general rule, no dangerous effects appear to have followed the respiration of this vapour for surgical purposes; but this inference has been chiefly drawn from those cases in which it had been administered for a very short period; and probably there was no tendency to congestion of the brain or lungs. In cases of prolonged respiration of the vapour, serious symptoms, and even death have resulted. . . . . . As in the case of all aërial poisons, the protracted respiration of ether vapour must tend to render recovery difficult, by thoroughly impregnating the blood with the poison. . . . . . The cause of death may be assigned partly to the want of aëration of the blood by oxygen, and its accumulation in this state in the brain; and partly to a directly poisonous action of the absorbed vapour, only manifested by its employment for a long period. The continual exhibition of morphia or strychnia, at intervals so short as not to allow of a recovery from each successive dose, must cause an accumulation in the system, and lead to fatal results. It is so with ether vapour, and experience now points to the propriety of withdrawing its use altogether in those cases in which the administration of it would require to be protracted for a long period." After citing several fatal cases of the administration of ether, the catalogue of which, by the bye, might be greatly extended, if dead men told tales, Mr. Taylor concludes : "These facts, then, show that the respiration of the vapour, even for so short a period as two minutes, may be in some instances attended with fatal consequences. In any case the inhalation of this vapour must be looked upon as temporary poisoning, with, *cæteris paribus*, a better chance of recovery than exists in most other instances of aërial poisoning."

These remarks of course apply with still greater force to chloroform, and are well worthy the attentive consideration of operators of surgery, and obstetric practitioners. They merit also the especial consideration of those who propose to employ anæsthetic agents in the treatment of maniacal affections.

